# Host–*Candida auris* interactions in the skin

**DOI:** 10.1371/journal.ppat.1014075

**Published:** 2026-03-27

**Authors:** Shankar Thangamani, Abishek Balakumar, Abhishek Datta, Garrett Bryak, Michail S. Lionakis

**Affiliations:** 1 Department of Comparative Pathobiology, College of Veterinary Medicine, Purdue University, West Lafayette, Indiana, United States of America; 2 Fungal Pathogenesis Section, Laboratory of Clinical Immunology and Microbiology, National Institute of Allergy & Infectious Diseases (NIAID), National Institutes of Health (NIH), Bethesda, Maryland, United States of America; Universitat Zurich, SWITZERLAND

## Abstract

*Candida auris* is an emerging, multidrug-resistant fungal pathogen that causes healthcare-associated outbreaks and life-threatening systemic infections. Unlike other *Candida* species, *C. auris* exhibits a distinct capacity for persistent skin colonization. In this review, we summarize our current understanding of clinical risk factors and host-microbe interactions that underlie *C. auris* skin colonization and infection. We discuss fungal determinants, including the unique mannan outer layer, fungal adhesins, the protein kinase Hog1, and other pathways in *C. auris* that govern adaptation in the skin. Furthermore, we highlight host immune mechanisms, including cytokine mediators (IL-1Ra, IL-17) and innate immune cells (neutrophils, macrophages, innate lymphocytes), that shape the outcome of *C. auris* skin colonization and infection. We also discuss how excessive IFN-γ responses drive epithelial pathology at the cutaneous barrier and enhance fungal persistence. Finally, we outline emerging research directions to understand host and microbe factors governing long-term colonization, with implications for developing novel therapeutic and vaccine strategies against this skin-tropic, multidrug-resistant fungal pathogen.

## Introduction

The emergence of *C. auris* has drawn global concern and has been designated by the US Centers for Disease Control and Prevention and the World Health Organization as an urgent threat and critical priority pathogen, respectively [1]. The six geographically stratified clades of *C. auris* are clade I (South Asian), clade II (East Asian), clade III (African), clade IV (South American), clade V (Iranian), and clade VI (Singapore) [2]. Among them, clades I, III, and IV are associated with antifungal resistance and a higher incidence of outbreaks [1,2]. Unlike most *Candida* species, such as *Candida albicans*, which colonize the gastrointestinal tract, *C. auris* exhibits a striking predilection for colonizing the human skin, enabling nosocomial transmission and outbreaks of fungemia and deep-seated infections [3–7]. In a recent taxonomic classification, *C. auris* was re-assigned to the genus *Candidozyma*, along with other closely related *C. haemuli* complex species [8]. Although the *C. haemuli* complex species are closely related to *C. auris*, they are still poor skin colonizers and do not cause outbreaks in hospitals [9]. Distinct from other skin-tropic fungi, such as *Malassezia* that remain confined to the epidermis [10], *C. auris* can invade the deeper dermis, a feature not previously observed among human commensal yeasts [3,11]. Moreover, skin surface colonization of the murine skin was shown to turn negative after 2 months, but live *C. auris* was recovered from deep skin tissue up to 4 months after initial colonization, suggesting that *C. auris* can persist in skin tissues for months, often evading detection during routine clinical surveillance [3,12,13]. Clade III (African) followed by clade IV (South American) had the highest colonization potential in murine skin tissue, with a long-term risk of persistent colonization [3]. Treatment is further complicated by widespread resistance to approved antifungal drugs [14,15]. Among antifungal agents, *C. auris* strains from the Mid-Atlantic regions are largely resistant to azoles and amphotericin B. Furthermore, healthcare isolates from the northeast, southeast, mountain, and west-coastal regions of the US are predominantly resistant to azoles [14]. The incidence of resistance to azoles and amphotericin B, and the emergence of pan-resistant isolates of *C. auris*, had increased in the US by 2021 [14]. The first instance of transmission of echinocandin resistance in *C. auris* was reported with the emergence of pan-resistant *C. auris* in US healthcare settings [16]. Because skin colonization facilitates *C. auris* transmission and hospital outbreaks, understanding the mechanisms governing fungal virulence at the cutaneous barrier is essential for preventing fungal colonization and identifying new therapeutic targets.

Fungal cell wall components constitute key pathogen-associated molecular patterns (PAMPs) that are recognized by the host to initiate and orchestrate antifungal immune responses, as previously reviewed [17]. Recent studies have shown that the *C. auris* cell wall is structurally and biologically distinct from that of *C. albicans* [18]. The outer mannan layer of *C. auris* consists of two unique Mα1-phosphate side chains not found in other *Candida* species [18]. Although anti-*C. albicans* host defense mechanisms have been well-characterized [17,19,20], they cannot be directly extrapolated to *C. auris* given these major cell wall differences between the species. A detailed understanding of *C. auris-*skin interactions is thus critical for elucidating its pathogenesis and informing the development of new antifungals and vaccines. This review summarizes recent advances in defining host, fungal, and microbiome factors that shape *C. auris* colonization, persistence, and pathogenesis at the skin barrier.

## Architecture of skin

**Fig 1:**
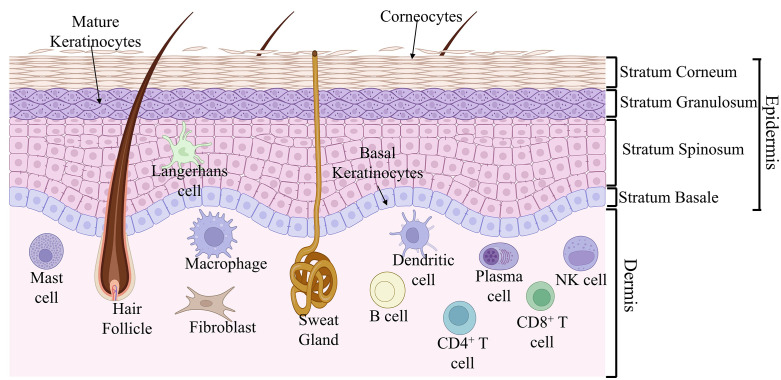
Architecture of the skin. Shown is the distribution of cells in the outer epidermis and inner dermis of the skin. The outer epidermis is composed of stratum corneum, consisting of corneocytes, stratum granulosum, consisting of mature keratinocytes, followed by stratum spinosum and stratum basale, consisting of basal keratinocytes. Langerhans cells are distributed within the epidermis, and the other cells, such as macrophages, dendritic cells, T cells, B cells, NK cells, mast cells, and fibroblasts, are distributed within the inner dermis. The figure was generated using licensed BioRender.

The skin is a dynamic anatomical barrier that protects the host from environmental microorganisms through its physical and immune defenses. It consists of two primary layers, the epidermis and dermis (Fig 1). The epidermis is subdivided into four layers: the stratum basale of continuously dividing undifferentiated keratinocytes; the stratum spinosum, where keratinocytes begin to mature and produce keratin; the stratum granulosum, in which keratinocytes produce lipids and keratin proteins: and the stratum corneum, the outermost layer composed of terminally differentiated, organelle-free corneocytes with crosslinked keratin fibrils [21–24]. This structure provides a unique physical barrier not found in other exposed epithelia, such as the respiratory or gastrointestinal mucosae [22–24]. Underneath the epidermis lies the fibrous dermis, which is composed of elastin and collagen and is subdivided into the superficial papillary and the deeper reticular dermis. The epidermis and dermis also contain skin appendages, including hair follicles, sweat glands, and sebaceous glands.

Beyond its structural role, the skin is an active immunological tissue populated by specialized immune and stromal cells that play a crucial role in host defense against invading pathogens. The epidermis harbors keratinocytes, Langerhans cells (LCs), and melanocytes, while the dermis contains macrophages, mast cells, fibroblasts, dermal dendritic cells (dDCs), T and B cells, innate lymphoid cells (ILCs), plasma cells, and NK cells (Fig 1) [23,25–28]. Keratinocytes express several pattern recognition receptors (PRRs), including surface (e.g., TLR1, 2, 4, 5, and 6) and endosomal (e.g., TLR3 and 9) Toll-like receptors (TLRs) [28]. Their activation leads to Th1-type immune responses and production of type I interferons (IFNs) [28–30] along with antimicrobial peptides (AMPs) such as β-defensins and cathelicidin [28], which are induced by IL-17 during infection (see section below) [28,31].

LCs, the principal antigen-presenting cells of the epidermis, express CD1a, langerin, and E-cadherin and contain Birbeck granules [32]. Upon antigen encounter and recognition via TLRs and C-lectin receptors (CLRs), LCs become activated and migrate to cutaneous lymph nodes to prime naïve lymphocytes [33]. dDC, the predominant antigen-presenting cell within the dermis, similarly captures and presents antigen after MHC class II upregulation. Macrophages, largely monocyte-derived in a CCR2-dependent manner [34], contribute to microbial uptake and clearance, cytokine production, and tissue homeostasis. Fibroblasts, though non-hematopoietic, also sense pathogens and secrete inflammatory mediators, highlighting the skin’s integrated immune-stromal cell network (see section below) [34–36].

## Predisposing clinical factors for *C. auris* skin colonization and infection

Certain patient conditions and treatments have been associated with an increased likelihood of *C. auris* skin colonization. These factors include metabolic disorders, diet, antimicrobials, immunosuppressive drugs, age, and prolonged hospitalization. Although a detailed understanding of the role of each factor in *C. auris* skin colonization and/or infection is lacking and is an important area for future study, evidence suggests that these factors, either directly or indirectly, contribute to an increased risk of *C. auris* infection in humans. For the purposes of this review, we define and use the terms “colonization and infection” as follows: The association of *C. auris* with the skin surfaces is defined as skin colonization. Studies using mouse or *ex vivo* skin models, with topical application or epicutaneous inoculation of *C. auris*, are considered models of *C. auris* colonization. On the other hand, studies using mouse models that employ intradermal or subcutaneous infection with *C. auris* are considered models of *C. auris* infection in this review.

### Metabolic disorders and diet

Surveillance studies across diverse geographical regions have identified diabetes as an underlying condition among patients with *C. auris* skin colonization (52% of cases) [37–39]; diabetes has also been repeatedly associated with an increased risk of *C. auris* systemic infection [15,40–43]. However, a study using topical application of *C. auris* in diabetic db/db mice, a widely used genetic model of type 2 diabetes, did not increase skin colonization [3], and susceptibility to *C. auris* skin colonization in chemically induced diabetic models, such as streptozotocin-treated mice, remains unexplored. Consuming a high-fat diet has been shown to alter skin architecture and local immune responses [33], which may help explain the association between obesity and *C. auris* infection in certain clinical studies [44]. Yet, short-term exposure to a high-fat western diet did not increase skin colonization by *C. auris* in mice [3]. Thus, longer-term or combined metabolic perturbations may better model human risk. Collectively, the contribution and mechanistic underpinnings of metabolic dysregulation and *C. auris* colonization and infection remain incompletely defined and warrant further investigation.

### Antibacterial agents

*C. auris* skin colonization frequently occurs in patients recently exposed to prolonged courses of broad-spectrum antibiotics, particularly carbapenems and vancomycin, with 43.3% of colonized patients treated with carbapenems, and 41.7% with vancomycin [40–43,45–47]. Antibiotics may facilitate fungal colonization by disrupting the skin microbiome and/or by dampening local host defenses. For example, vancomycin has been shown to promote *C. auris* growth *in vitro* and in a *Galleria mellonella* infection model [48] and to impair lymphocyte-dependent IL-17 responses at mucosal barriers [49]. Broad-spectrum antibiotic use has also been shown to alter the skin microbiome composition and to enrich for multidrug-resistant organisms such as *Pseudomonas aeruginosa*, *Proteus mirabilis*, *Acinetobacter baumannii*, *Klebsiella pneumoniae*, and *Providencia stuartii*, potentially creating a permissive microbial niche for *C. auris* [12,13]. Jo and colleagues demonstrated that systemic antibiotic administration, such as doxycycline and trimethoprim/sulfamethoxazole (TMP/SMX), can penetrate the dermis and epidermis, altering the skin bacterial microbiome and enriching antibiotic-resistant strains [50]. This alteration in the microbiome may create a permissive environment for *C. auris* to colonize. In mice, pre-exposure to select antibiotics alone (tetracycline, trimethoprim-sulfamethoxazole) or in combination (ampicillin, metronidazole, neomycin, vancomycin [AMNV]) did not affect *C. auris* skin colonization [3]. These findings underscore interspecies differences in microbiota composition. *Staphylococcus* species such as *S. epidermidis*, *S. hominis*, and *S. capitis* are specific to human skin, whereas *S. saprophyticus*, *S. aureus*, and *S. lentus* are predominant species in mice [51,52]. This highlights the need for additional human-based studies to delineate the mechanisms by which microbial dysbiosis may influence *C. auris* persistence or colonization resistance.

### Antifungal agents

The use of antifungals can alter or decrease the abundance of the skin’s mycobiome. A previous study shows that the human skin mycobiome regulates *C. auris* colonization in the skin [12]. Prior use of antifungals such as fluconazole has been associated with an increased risk for *C. auris* skin colonization and infection, which represented 11.7% of the cases [45] and 65.2% of the invasive infections in humans [53]. These findings suggest that exposure to antifungals may be a possible risk factor for *C. auris* colonization and infection in hospitalized patients. However, the human subjects in these studies had pre-existing comorbidities and medical conditions.

### Immunosuppressive drugs

Immunosuppressive medications, especially corticosteroids, have been reported to be a predisposing factor for *C. auris* colonization. Studies assessing potential risk factors of *C. auris* show that ~35% of patients who received corticosteroids were associated with *C. auris* colonization [42,54] It is likely that suppression of IL-17-mediated responses is critical for cutaneous antifungal defenses [3,55] and/or inhibition of other innate and adaptive antifungal immune responses [56]. Cyclophosphamide-induced neutropenia increased mortality in mouse models of systemic *C. auris* infection [57,58], suggesting that immunosuppression augments susceptibility to *C. auris* colonization and dissemination, as has also been observed with other *Candida* species [59].

### Age

The skin barrier function weakens with age, and the microbiome composition changes significantly across age groups. *Proteobacteria* and *Corynebacterium* were reported to be the predominant phylum and genus, respectively, on the skin with aging [60,61]. The skin of older individuals also shows reduced lipid content and natural moisturizing factors, which are essential for maintaining an effective stratum corneum barrier [62,63]. The age of the individual has been reported as a predisposing factor for *C. auris* skin colonization in some studies, with *C. auris*-colonized patients aged 65–80 years at greater risk [62–64].

### Prolonged hospitalization

*C. auris* demonstrates a remarkable ability to persist on both host skin and inanimate objects for extended periods of time, posing great challenges to eradicate from hospital settings. Notably, many hospital-grade disinfectants have limited efficacy against *C. auris* [12,64,65]. As a result, patients with long-term hospital stays, especially those in long-term acute care hospitals (LTACHs) [66,67], were found to be likely to have *C. auris* skin colonization.

### Other clinical risk factors

Additional clinical conditions associated with *C. auris* colonization and/or systemic infection include chronic kidney disease, hemodialysis, malignancy, central venous or urinary catheterization, and total parenteral nutrition, mirroring known risk factors for infections by other *Candida* species [45,46,59]. How these comorbidities, individually or collectively, increase the risk of *C. auris* skin colonization and infection remains to be studied.

## Microbiome factors that regulate *C. auris* skin colonization

As a skin-tropic pathogen, *C. auris* must engage in complex interactions with the resident skin microbiome. A successful colonization is possible when existing microbial communities are permissive to its presence, rather than excluding it. The currently known associations between various bacterial microbiome communities and the presence or absence of *C. auris* colonization are discussed below.

### Bacterial microbiome

**Fig 2:**
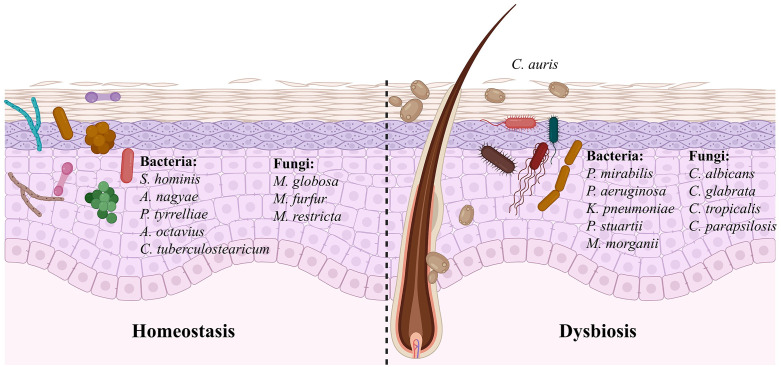
Microbiome alterations in *Candida auris*-positive and negative human skin. Shown are bacterial and fungal communities distributed on the skin during normal homeostasis and during dysbiosis, which has been associated with *C. auris* skin colonization. The figure was generated using licensed BioRender.

A point prevalence survey conducted by Proctor and colleagues in a ventilator-capable skilled nursing facility in Chicago showed that patients colonized with *C. auris* on their skin harbor a distinct skin bacterial microbiome compared with uncolonized individuals from the same facility [12]. Patients with *C. auris* skin colonization were frequently co-colonized with multidrug-resistant gram-negative bacteria, including *Proteus mirabilis, Pseudomonas aeruginosa, Klebsiella pneumoniae, Providencia stuartii,* and *Morganella morganii* (Fig 2) [12,13]. In contrast, patients who were not colonized with *C. auris* harbored a distinct set of bacteria, such as *Staphylococcus hominis*, *Staphylococcus caprae*, *Anaerococcus nagyae*, *Peptoniphilus tyrrelliae*, *Anaerococcus octavius*, *Corynebacterium tuberculostearicum*, and *Staphylococcus epidermidis* (Fig 2) [12]. These findings suggest that *C. auris* colonization is associated with distinct bacterial communities, potentially reflecting either ecological compatibility or loss of colonization resistance. Whether this cohabitation is mutualistic or opportunistic remains unclear. Future studies employing animal models and longitudinal human sampling are needed to define bacterial taxa that modulate *C. auris* persistence and identify mechanisms of microbial interference at the skin barrier.

### Fungal mycobiome

Several fungal species constitute a significant proportion of the skin microbiome, termed the mycobiome, which may potentially influence *C. auris* colonization [68]. Proctor and colleagues identified four distinct mycobiome clusters in patients, distinguished by the relative abundance of *Malassezia* and *Candida* species [12]. Skin samples enriched for *Malassezia* species, including *M. globosa*, *M. furfur*, *M. restricta*, and *M. arunalokei,* which dominate the healthy human skin mycobiome, were rarely positive for *C. auris* [12,69]. Conversely, *C. auris* abundance correlated with other *Candida* species such as *C. tropicalis*, *C. parapsilosis*, *C. orthopsilosis,* and *C. metapsilosis* (Fig 2) [12]. Similarly, *C. auris*-positive dog ears exhibited reduced abundance of *Malassezia* species [70]. These observations suggest that *Malassezia* species may confer colonization resistance, whereas coexisting *Candida* species may create a permissive niche for *C. auris.* Since skin microbial communities can restrict bacterial pathogens through metabolic competition and immune modulation [69–72], dissecting inter-fungal and bacterial-fungal interactions will be essential for a better understanding of *C. auris* colonization and for developing microbiome-based preventive and therapeutic strategies.

## Fungal factors that regulate *C. auris* skin colonization

Recent studies have identified several *C. auris* virulence factors that underlie its propensity for skin colonization. Although fewer than 1% of *C. auris* open reading frames have been functionally characterized [71], emerging work has highlighted key roles for adhesins, protein kinases, and transcription regulators in mediating adhesion, biofilm formation, and adaptation to environmental stress in *C. auris*.

### Adhesins: Scf1, Iff4109, and Als4112

By performing an elegant forward genetic screen of insertional mutants in *C. auris*, Santana and colleagues identified a novel adhesin, Scf1 (surface colonizing factor), which promotes fungal adhesion to inert abiotic surfaces through cation-dependent interactions and enhances biofilm formation on murine and human skin [6]. *SCF1* functions redundantly with the adhesin *IFF4109*, a member of the IFF/HYR adhesin family, as their double deletion in the *Δscf1* Δ*iff4109* mutant strain significantly decreased skin colonization. Notably, *SCF1* expression varies between *C. auris* clades and within strains of the same clade and correlates with adhesive capacity; thus, SCF1 expression is low in the poorly adherent strain AR0387 and elevated in the highly adherent strain AR0382. Furthermore, overexpression of *SCF1* in the poorly adherent strain AR0387 restored its adherence capacity and increased its colonization potential in murine and human skin [6].

Als4112, a member of the agglutinin-like sequence (Als) adhesin family, further enhances *C. auris* cell aggregation and biofilm formation [72,73]. Clinical isolates of *C. auris* exhibiting aggregative phenotypes often harbor amplification of the sub-telomeric *ALS4112* locus, conferring robust biofilm formation and colonization on murine skin [73]. Als4112 physically interacts with Scf1 through the Flo11 and serine–threonine-rich domains, and together these adhesins mediate in cell-cell adherence, aggregation, and biofilm integrity for skin colonization [74].

*C. auris* colonizes both superficial and deep skin layers and can reside within hair follicles that are lined by keratinocytes [3]. A recent study performed unbiased insertional mutant screening in *C. auris* and confirmed that *ALS4112* mediates keratinocyte adherence [11]. *ALS4112* deletion abolished the adherence capacity on human keratinocytes across strains from multiple *C. auris* clades, while its overexpression enhanced adherence in the poorly-adherent strain AR0382 [11]. Concordantly, among *C. auris* clinical isolates, the magnitude of *ALS4112* expression correlated with their keratinocyte adherence capacity, with Clade IV exhibiting comparatively higher adherence. Moreover, it was recently shown that *C. auris* binds avidly to extracellular matrix (ECM) proteins, such as tropoelastin, collagen V, and laminin, with Als4112 mediating binding to the principal ECM protein, laminin [11].

The skin colonization potential varies among the different geographically stratified clades of *C. auris* [3]. This shows that differential fungal factors and their gene expression across clades may contribute to variable propensities for *C. auris* skin colonization. Recent studies suggest that the gene expression of *SCF1* and *ALS4112* varies among different clades and clinical strains of *C. auris*, and exhibits a strong positive correlation with *C. auris* adherence activity [6,11]. *C. auris* strains belonging to clade III highly express *SCF1* and *ALS4112,* associated with greater adherence capacity. On the other hand, clade II had low expression of *SCF1* and *ALS4112,* and exhibited low adherence capacity. Furthermore, strains belonging to clades I and IV uncoordinatedly express *SCF1* and *ALS4112,* with regulation independent among the individual strains. Moreover, deletion of *Δscf1*Δ*iff4109* and *ΔAls4112* significantly reduced skin colonization, suggesting a vital role of these adhesins in *C. auris* colonization of the skin [6,11]. This suggests that the gene expression of *SCF1* and *ALS4112* across *C. auris* clades and strains may affect their skin colonization potential. Collectively, adhesins Scf1, Iff4109, and Als4112 are key effectors that enable *C. auris* attachment to keratinocytes and the ECM, providing a molecular basis for understanding its skin tropism and representing potential targets for antifungal drug development*.*

### Protein kinase Hog1

The protein kinase Hog1 governs *C. auris* adaptation to host-imposed stress conditions [75,76]. Specifically*,* Hog1 confers resistance to osmotic, cationic, acid, and reactive oxygen species-induced stress [75,76]. A *HOG1*-deficient mutant strain exhibited reduced epidermal colonization, intradermal persistence, and dissemination to distant organs following epicutaneous and intradermal infections in mice [76]. Similarly, during systemic murine infection, fungal proliferation in the kidney and brain was significantly decreased in the *Δhog1* mutant strain [76]. Thus, Hog1-dependent stress tolerance is critical for both cutaneous and systemic spread of *C. auris*.

### Other pathways of *C. auris* governing stress tolerance and metabolic adaptation in the skin

*C. auris* can proliferate in the nutrient-poor environment of the skin, in which other pathogenic fungi fail to persist long-term. In recent years, additional pathways governing stress tolerance and *C. auris* metabolic adaptation in the skin have been explored to understand how *C. auris* remodels its metabolism and activates survival pathways distinct from those of other *Candida* species. Sebum and sweat, which are rich in free fatty acids and amino acids, are the major nutrients of the skin. In nutrient-poor environments lacking glucose, *C. auris* utilizes free fatty acids via β-oxidation, followed by the glyoxylate cycle to obtain energy from the carbon source. Recent evidence suggests that deletion of *FOX2* (β-oxidation), *CAT2* (fatty acid transport), and *ICL1* (glyoxylate cycle) results in defective growth in sweat media [77]. This metabolic adaptation of *C. auris* may favor long-term persistence in the skin.

*C. auris* further regulates other stress resistance pathways, such as calcineurin [78] and Ras/cAMP/PKA [79] pathways for thermotolerance, morphological plasticity, and biofilm formation, which contribute to pathogenesis in *C. auris*. Cha and colleagues demonstrated that ablation of the calcineurin pathway by *CNA1* deletion in *C. auris* significantly reduced abscess and fungal burden in the murine skin in a subcutaneous infection model [78]. In *ex vivo* murine skin, the *Δcna1* strain had significantly lower adhesion to the skin shortly post-association [78]. This suggests that the *CNA1* is indispensable for virulence properties needed for *C. auris* skin colonization and infection [78]. Furthermore, hyperactivation of the Ras/cAMP/PKA pathway by *BCY1* or *PDE2* deletion attenuated the virulence during murine systemic infection by reducing the ability to thrive in nutrient-deficient conditions [79,80]. However, the precise mechanisms by which these pathways contribute to *C. auris* persistence in the skin remain unclear and require further investigation.

## Innate immune defense mechanisms against *C. auris* in the skin

Innate immune responses in the skin are orchestrated by several immune cells, including neutrophils, macrophages, and ILCs. Non-immune cells can also play an important role in innate defense by contributing to skin barrier integrity and secreting antimicrobial peptides to defend against invading pathogens. Innate antifungal immune mechanisms against other fungal pathogens have been reviewed in detail elsewhere [19,81]. Here, we specifically discuss innate mechanisms relevant to *C. auris* and contrast them with their known roles in *C. albicans*.

### Neutrophils are critical for skin immunity against *C. auris*

Neutrophils are critical in host defense against several fungal pathogens, yet their role in cutaneous immunity to *C. auris* in the skin has only recently been defined [82,83]. Unlike *C. albicans, C. auris* exhibits remarkable resistance to neutrophil phagocytosis and killing *ex vivo*, attributable to its unique cell wall composition [18,84]. Johnson and colleagues reported that primary human neutrophils show reduced phagocytosis and impaired neutrophil extracellular trap (NET) formation against *C. auris ex vivo* compared to *C. albicans* [84]. Using a zebrafish model of invasive candidiasis, reduced neutrophil recruitment *in vivo* was observed during *C. auris* compared to *C. albicans* infection [84]. Similarly, in an intradermal mouse model of *C. auris* infection, neutrophil accumulation was significantly lower in *C. auris*-infected compared to *C. albicans*-infected mice *in vivo* [85]. Despite the blunted neutrophil accumulation during *C. auris* infection, antibody-mediated neutrophil depletion in mice significantly increased fungal burden in the skin *in vivo*, establishing neutrophils as a contributing factor to anti-*C. auris* skin defense, albeit being functionally subverted during infection [86].

Recent work linked this neutrophil evasion to *C. auris* cell wall mannosylation [87]. CRISPR-mediated deletion of *PMR1* and *VAN1*, genes required for N-mannan synthesis and branching, significantly reduced cell wall mannan content and reciprocally increased exposure of β-glucan and chitin [87]. These mutant strains exhibited improved recognition and killing by human neutrophils *ex vivo*, and their inoculation into the zebrafish model of invasive candidiasis resulted in increased neutrophil recruitment, associated with reduced fungal burden [87]. Correspondingly, wild-type *C. auris* resisted neutrophil killing, whereas deletion of *PMR1* and *VAN1* in *C. albicans* and *C. glabrata* did not alter their engagement with neutrophils, thereby highlighting a species-specific role for *C. auris* mannosylation in neutrophil evasion [87].

scRNA-seq of *C. auris* murine skin during infection identified *IL1RN* (encoding IL-1 receptor antagonist, IL-1Ra) as a key host factor modulating neutrophil activity during *C. auris* infection [86]. IL-1Ra is produced predominantly by macrophages in response to *C. auris* and inhibits IL-1α and IL-1β signaling by competitively binding to IL-1R [88,89]. Exogenous IL-1Ra decreased the killing capacity of neutrophils *ex vivo* against *C. auris*, while IL-1Ra neutralization in *C. auris*-infected mice *in vivo* significantly reduced cutaneous fungal burden, demonstrating that excessive IL-1Ra dampens protective neutrophil responses and favors fungal growth in the skin. Notably, *ex vivo* macrophages primed with wild-type *C. auris*, but not the *pmr1Δ* mutant strain lacking outer mannan, produced elevated IL-1Ra, linking the fungal mannan layer to the IL-1R pathway inhibition. In turn, IL-1R deficiency impaired neutrophil recruitment even against the *pmr1Δ* mutant strain, confirming the requirement of IL-1 signaling for anti-*C. auris* antifungal immunity in the skin.

Together, these findings uncover a two-tiered evasion strategy: *C. auris* masks immunogenic β-glucan through mannosylation to avoid neutrophil recognition and simultaneously induces macrophage-derived IL-1Ra to suppress IL-1R-dependent neutrophil recruitment and activation. This mannan-IL-1Ra axis represents a critical checkpoint exploited by *C. auris* to persist in the skin and a potential therapeutic target.

### Macrophages are associated with increased skin fungal burden

Macrophages are pivotal in host defense against several fungal pathogens, yet during *C. auris* skin infection appears to facilitate, rather than restrict, fungal persistence [81]. Flow cytometric and scRNA-seq analyses revealed increased accumulation of macrophages at the site of *C. auris* skin infection in mice [86], but their functional role remained poorly defined. Recent studies showed that *C. auris* can replicate within macrophages, using metabolic plasticity to survive and escape the phagolysosomal environment [90,91]. The fungus induces host glucose starvation and metabolic stress, leading to macrophage death without triggering classical activation of the NLRP3 inflammasome or its effector molecules IL-1β and caspase-1, unlike *C. albicans* [90]. Disruption of mitochondria and iron homeostasis restricts the proliferation of *C. auris* within macrophages, underscoring the metabolic dependence of this phenomenon [91].

*In vivo*, macrophage depletion in murine *C. auris* skin infection markedly reduced fungal burden. This protective effect likely reflects macrophage-derived IL-1Ra and increased neutrophil antifungal activity, though direct deprivation of an intracellular replication niche may also contribute. Future studies should define how *C. auris* modulates macrophage metabolism and viability during skin infection, and whether its distinctive mannan layer of *C. auris* conceals fungal PAMPs from inflammasome detection and activation.

### Essential role of ILCs against *C. auris* in the skin

ILCs contribute to effective host defense against mucocutaneous fungal challenge [92,93]. In an intradermal mouse model, IL-17^+^ ILCs accumulated in the skin during *C. auris* infection [85]. Similarly, Huang and colleagues demonstrated in an epicutaneous model that both Rag2-deficient mice, which lack T cells but retain ILC populations, as well as Rag2 Il2rg double-knockout mice, which lack ILCs in addition to all other lymphoid cells, were highly susceptible to *C. auris* skin colonization [3]. Susceptibility was greatest in Rag2 Il2rg double-knockout mice, confirming that IL-17A/IL-17F-producing lymphoid populations, including but not limited to ILCs, are essential for protection against *C. auris* skin colonization [3].

The type-17 cytokine, IL-22, although critical for protection against *C. albicans* oral infection, appears dispensable for immunity in the setting of skin *C. auris* colonization [3,94]. These findings highlight distinct cytokine requirements across mucocutaneous tissues and underscore IL-17-producing ILCs as key effectors that maintain antifungal barrier immunity in the skin.

### Role of non-immune skin cells in antifungal immunity to *C. auris*

Non-immune skin cells contribute to barrier defense by maintaining epithelial integrity, producing AMPs, and releasing chemokines to recruit immune effector cells [95,96]. Thus, keratinocytes, fibroblasts, and endothelial cells together form a dynamic frontline against microbial invasion.

Dermal fibroblasts are central among those populations. During *S. aureus* subcutaneous infection in mice, dermal fibroblasts undergo reactive adipogenesis and expand as preadipocytes that secrete CAMP (Cathelicidin Antimicrobial Peptide) [97]. A similar fibroblast-to-preadipocyte transition has been observed during *C. albicans* intradermal infection of mice, where CAMP production by preadipocytes is regulated through the FGFR-MEK-ERK signaling pathway [98]. Utilizing an intradermal mouse model of *C. auris* skin infection, scRNA-seq identified a fibroblast subset expressing adipocyte lineage markers as the only proliferating non-immune cell population [83], suggesting an analogous reactive adipogenesis during cutaneous *C. auris* infection. Skin fibroblast subsets also expressed a broad array of cytokines, chemokines, PRRs, and AMPs in response to *C. auris*, including *Il33*, *Csf1*, *Tgfb2, Ccl2, Ccl7, Ccl11, Cxcl1, Cxcl5, Cxcl9, Cxcl10, Cxcl12, Cxcl14, Clec3b, Clec11a, Nod1, Mrc2, Lgals1, Lgals9, Lman1, Lnc2, Adm* and *Ang* [86]. These mediators likely coordinate neutrophil, monocyte, and macrophage recruitment and promote local antimicrobial activity during *C. auris* skin infection.

**Fig 3:**
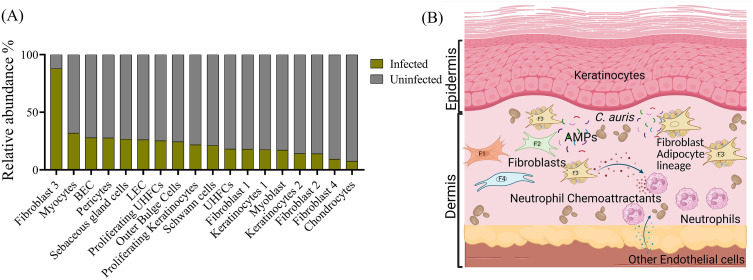
Non-immune cells and *Candida auris* skin infection. **(A)** Non-immune cells accumulated during *C. auris* skin infection *in vivo* as identified by single-cell RNA sequencing data generated in [86]. **(B)** Potential involvement of stromal cells, such as fibroblast subsets and adipocytes, in regulating immune response during *C. auris* skin infection by production of AMPs and neutrophil chemoattractants. The figure was generated using licensed BioRender. Abbreviations: BEC, Blood endothelial cells; LEC, Lymphatic endothelial cells; UHFCs, Upper hair follicle cells; AMPs, Antimicrobial peptides; F1, Fibroblast 1; F2, Fibroblast 2; F3, Fibroblast 3; F4, Fibroblast 4.

Endothelial cells, likewise, upregulate several neutrophil chemoattractant molecules during *C. auris* skin infection, likely supporting neutrophil extravasation and recruitment from the blood to the infected tissue. A broader spectrum of cutaneous stromal cells was also identified with a dynamic transcriptional program during *C. auris* infection (Fig 3). Together, these findings demonstrate that non-immune stromal cells mount a coordinated innate response during *C. auris* infection, coupling structural maintenance with immune activation. Future *in vivo* studies using cell-specific conditional knockout mice will be critical to define how keratinocyte-, fibroblast-, and endothelium-derived mediators influence fungal containment during *C. auris* infection.

## Adaptive immunity in skin during *C. auris* encounter

IL-17-mediated immunity is central to mucocutaneous host defense against fungi such as *C. albicans* and *Malassezia* [10,99,100]. Adaptive immune responses during *C. albicans* and *Malassezia* are discussed in detail elsewhere [10,20]. In this review, we focus on adaptive immune responses to *C. auris* and on how IL-17 and IFN-γ play opposing roles in regulating *C. auris* colonization and infection of the skin.

### Protective role of IL-17 in skin against *C. auris*

**Fig 4:**
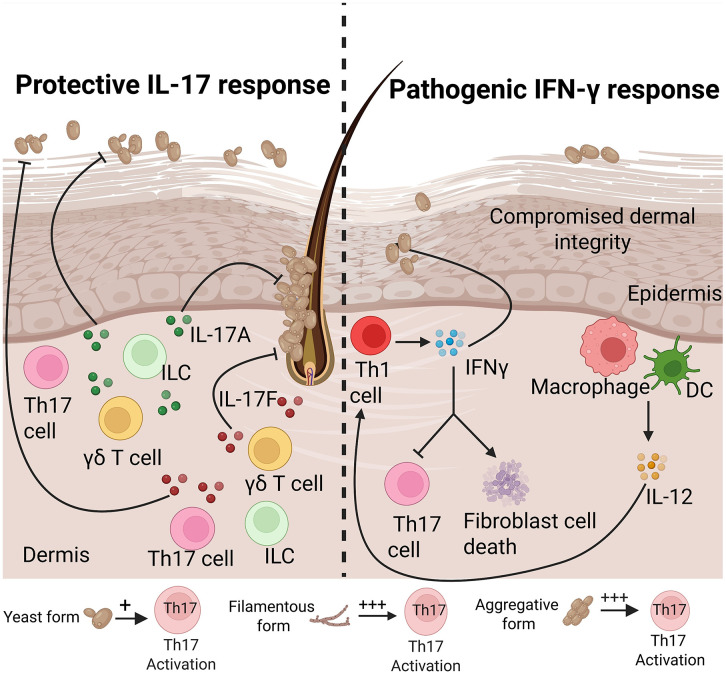
Protective role of IL-17 and pathogenic role of IFN-γ during *Candida auris* skin infection. IL-17 produced by Th17 cells, γδ T cells, or ILCs in response to *C. auris* in the skin plays a protective role in host defense. IL-12 induced by macrophages and dendritic cells during *C. auris* interaction triggers Th1 cells to produce IFN-γ, which compromises dermal integrity and induces fibroblast cell death. The yeast, filamentous, and aggregative forms of *C. auris* can induce differential magnitudes of Th17 responses. The figure was generated using licensed BioRender.

In both epicutaneous colonization and intradermal infection mouse models, *C. auris* induces the accumulation of CD4^+^ IL-17A^+^ and CD4^+^ IL-17F^+^ Th17 cells in the skin [3,85]. Using Act1-deficient mice lacking IL-17 receptor signaling, Huang and colleagues showed that IL-17A/IL-17F responses are critical for cutaneous protection against *C. auris* colonization (Fig 4) [3]*.* As noted above, Rag2-deficient mice display similarly increased susceptibility to *C. auris* skin colonization, indicating that both αβ T cells and γδ T cells are crucial for IL-17-dependent immunity in this model. Future studies using CD4-, CD8-, TCRαβ-, and TCRγδ-deficient mice will be essential to delineate the relative contributions of Th17 cells, Tc17 cells, and IL-17-producing γδ T cells to cutaneous defense against *C. auris*.

In contrast to *C. albicans*, a deficiency in the CLR adaptor Card9, required for the induction of Th17 cells during cutaneous *Malassezia* infection [10,101,102], did not affect *C. auris* control in the skin [3]. Moreover, whereas LCs promote the induction of Th17 cells during *C. albicans* skin infection [103], LC-deficient mice controlled *C. auris* colonization normally, collectively indicating that *C. auris* elicits protective IL-17 immunity through Card9- and LC-independent pathways during colonization of the skin [3]. Notably, *C. auris* induces a weaker Th17 cell response than *C. albicans*, which may contribute to its long-term persistence [85]. Defining the fungal determinants that regulate Th17 responses during the *C. auris*-skin interaction remains a key area for investigation.

### Fungal morphology shapes Th17 immunity

Fungal morphology profoundly influences virulence and host immunity [103]. In the skin, *C. albicans* exists as a yeast form in the stratum corneum, whereas in the dermis and systemic organs, it predominantly exists as filamentous forms. Of note, the yeast-locked form of *C. albicans* induces a robust Th17 response [103]. Clinical isolates of *C. auris* exhibit distinct morphologies, including yeast, aggregative, and filamentous [104–108]. To investigate the skin immune response against yeast and filamentous forms of *C. auris*, deletion of *ELM1*, a negative regulator of filamentation, was employed to convert the wild-type South Asian *C. auris* strain AR0387 to a filamentous strain; this elicited markedly greater Th17 cell responses and reduced fungal burden in murine intradermal skin infection [104]. Correspondingly, complementation restored yeast morphology and baseline Th17 responses [104]. These findings, consistent with prior *C. albicans* studies [86], demonstrate that *C. auris* morphotypes qualitatively and quantitatively regulate IL-17 immunity and fungal burden in skin.

Additional studies recently examined how the aggregative phenotype of *C. auris,* a complex phenomenon controlled at least in part by the zinc-finger transcription factor *ACE2* that governs cell separation via the chitinase *CST1*, regulates Th17 cell development [109,110]. As such, cells lacking *ACE2* fail to separate following budding and remain clumped together or “aggregate”, becoming resistant to physical stress and disruption [111]. Recent reports showed that clinical isolates of *C. auris* that harbor ACE single-nucleotide polymorphisms form rough, adherent colonies [112]. In a murine model of intradermal infection, the *ace2Δ* mutant strain of *C. auris* induced greater Th17 responses compared to wild-type or complemented strains [109]. Collectively, yeast, filamentous, and aggregative forms of *C. auris* elicit distinct magnitudes of Th17 activation, likely reflecting differences in cell wall composition and surface antigen exposure (Fig 4) [87,113].

These observations reveal a link between *C. auris* morphogenesis and adaptive immunity, suggesting that morphotype-specific cues may determine the quality and strength of protective IL-17 responses. Elucidating how *C. auris* morphology shapes T helper cell polarization and effector responses will be critical for designing antifungal vaccines or immunotherapies that restore effective skin immunity.

### The pathogenic role of IFN-γ during *C. auris* skin infection

Whereas type-17 responses are protective in cutaneous antifungal defense, the contribution of type-1 immunity remained unclear during *C. auris* interaction with the skin [103,114]. Recent studies have shown that *C. auris* infection preferentially induces IFN-γ-secreting pathogenic Th1 cells upon re-infection (Fig 4) [115,116]. IFN-γ exacerbates skin infection by *C. auris* but not *C. albicans* via two independent mechanisms [115]*.* First, *Ifng-/-* mice displayed lower fungal burden associated with increased Th17 cells and expression of IL-17 receptor-dependent genes such as *Cxcl1, S100a8, Il6*, and *Il1b*. These findings suggest that excess IFN-γ partially attenuates protective IL-17-mediated responses following secondary infection with *C. auris,* thereby promoting persistence*.* Secondly, IFN-γ drives apoptosis of epithelial cells and fibroblasts and disrupts barrier integrity [117–119]. As such, wild-type mice exhibited higher frequencies of dead EpCAM^+^ epithelial cells and PDGFRα^+^ fibroblasts than *Ifng−/−* mice, and IFN-γ exposure caused dose-dependent cell death in primary keratinocytes and fibroblasts *in vitro,* particularly following *C. auris* challenge [115]. Consistently, keratinocyte-specific IFNγR1 conditional knockout mice were protected from barrier damage and fungal proliferation and persistence *in vivo* [116]. These findings were also corroborated in the context of IL-17 deficiency, thereby uncoupling the detrimental effects of excessive IFN-γ from its indirect effects on IL-17 responses and demonstrating that IFN-γ directly injures epithelial barriers, thereby enhancing *C. auris* colonization [116]. These findings extend a broader paradigm [120], which was first established in autoimmune polyendocrinopathy-candidiasis-ectodermal dystrophy (APECED) syndrome caused by loss-of-function variants in the autoimmune regulator (*AIRE*) gene, in which chronic excessive IFN-γ-driven mucosal inflammation promotes chronic mucocutaneous candidiasis by disrupting the integrity of the oral epithelial barrier [117,121].

At the cellular level, IL-12-producing inflammatory macrophages and monocyte-derived dendritic cells are the key sources of IFN-γ-inducing signals during *C. auris* re-infection (Fig 4). Indeed, depletion of these populations with clodronate liposome or *Ccr2−/−* mice decreased IL-12 production and Th1 cell development *in vivo* [122]. Given that the host immune response is modulated by fungal PAMPs [17] and that *C. auris* features a structurally unique outer mannan layer [18], that study also examined whether this distinct cell wall structure modulates Th1 cell development. Infection with the *C. auris* mannosylation-deficient *pmr1Δ* mutant strain resulted in decreased Th1 and increased Th17 cells, indicating that the *C. auris* outer mannan layer skews adaptive immunity toward type-1 polarization to promote fungal persistence in the skin [115].

Together, these findings define excessive IFN-γ as a pathogenic amplifier that damages epithelial barriers, suppresses Th17 responses, and favors chronic *C. auris* infection in the skin (Fig 4) [115]. This mechanistic insight suggests that selective modulation of type-1 cytokine pathways, such as with IFN-γ or IL-12 blockade, may represent a novel therapeutic approach to restore skin immunity and prevent persistent *C. auris* infection in vulnerable patients.

## Conclusions

*C. auris* has redefined the landscape of fungal pathogenesis as an emerging, multidrug-resistant, skin-tropic microbe capable of long-term colonization, transmission, and life-threatening invasive disease. Unlike *C. albicans,* which primarily colonizes mucosal surfaces, *C. auris* uniquely persists within the skin and hair follicles, an ecological niche where fungal, host, and microbiome factors intersect. Studies over the past decade now delineate a distinct immunological signature of *C. auris* infection characterized by impaired neutrophil recruitment, IL-1Ra-mediated immune suppression, detrimental macrophage responses, and an imbalance between protective type-17 and pathogenic type-1 cytokine circuits. These findings collectively establish *C. auris* as a model to understand how cytokine polarization, epithelial integrity, and fungal evolution converge to shape the balance between protective and detrimental cutaneous immunity.

Future work is needed to help integrate microbiome, fungal, and host genetics to dissect mechanisms of colonization resistance and immune evasion. For example, the observation that *C. auris* coexists with specific bacterial communities suggests that probiotic commensal interventions, akin to ongoing clinical trials using *Staphylococcus hominis* or *Roseomonas mucosa* against *Staphylococcus aureus* and/or atopic dermatitis [123–125], could be leveraged to competitively inhibit *C. auris* skin colonization. In parallel, dissecting fungal antigens and effector T cell responses will be essential for rational vaccine design. While the NDV-3A, based on *C. albicans* Als3 protein, provides partial protection against systemic *C. auris* infection in mice [126], its role in protecting against skin colonization and infection remains unclear; thus, further defining the tissue-resident memory T cell and cytokine requirements unique to *C. auris* in the skin will be crucial for developing next-generation antifungal vaccines.

Finally, understanding immunopathology at the skin barrier, particularly IFN-γ-driven epithelial damage, may help guide host-directed therapeutic strategies that restore protective immunity without exacerbating inflammation. As multidrug resistance in *C. auris* currently limits antifungal drug options, targeting host pathways that maintain barrier integrity, modulate cytokine balance, and/or exploit commensal competition represent forward-looking strategies to prevent skin colonization and systemic infection. Therefore, while *C. auris* is an emerging global public health threat, it also serves as an experimental framework for linking basic discovery to translational interventions that will safeguard barrier immunity in humans.
